# Supplementary material for: In Vitro Hypocholesterolemic Effect of Coffee Compounds
Source: Nutrients. 2020 Feb 9;12(2):437. doi: 10.3390/nu12020437 (PMC7071201; doi:10.3390/nu12020437)
Supplement: Supplementary file 1 [file nutrients-12-00437-s001.pdf]

Supplementary material

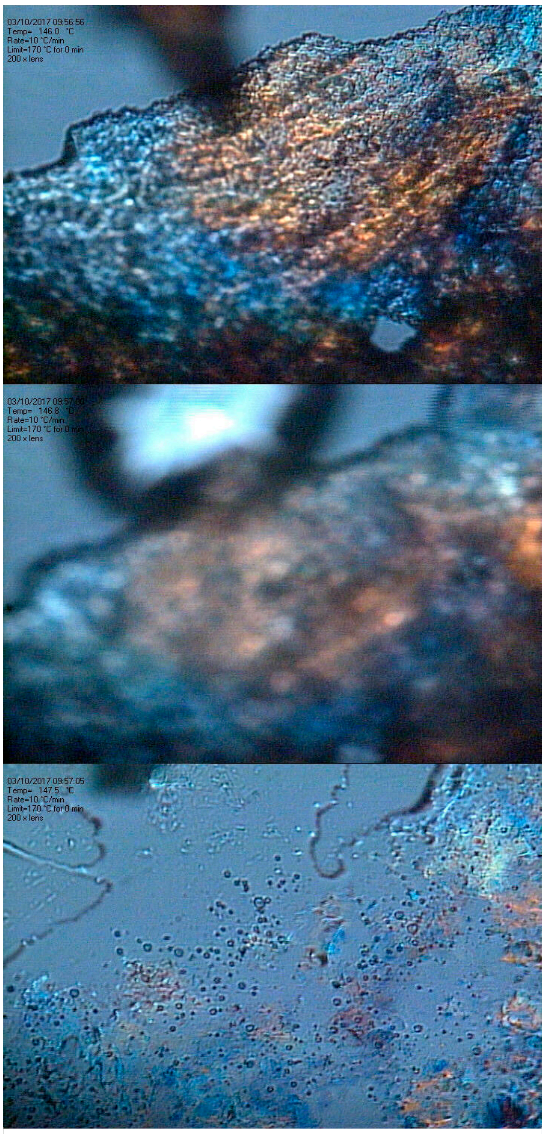

Figure S1. Polarized light thermal microscope photograph showing the transition temperature observed for the crystals obtained in the intestinal model containing coffee extracts
